# A Case Study on the Critical Role of Advance Care Planning

**DOI:** 10.14740/jmc5239

**Published:** 2026-02-02

**Authors:** Sarah Portnell, Aryan Patel, Linda Akbarshahi, Catherine Kuo, Hardeep Singh

**Affiliations:** a GME Research, Northeast Georgia Medical Center, Gainesville, GA, USA; b Family Medicine, Northeast Georgia Medical Center, Gainesville, GA, USA

**Keywords:** Advance care planning, Resuscitation orders, Medical power of attorney, Life-sustaining treatments

## Abstract

Advance care planning (ACP) is a vital process that allows patients to express healthcare desires before they are unable to make decisions on their own behalf. Despite its guidance in end-of-life care, nearly 30% of patients die without having clearly articulated their goals, leading to uncertain decision-making and potential unwanted medical interventions. ACP incorporates documents such as living wills, medical power of attorneys (POAs), and physician orders for life-sustaining treatment (POLST). These forms provide a structured format for patients, mainly those with chronic illness or aging, to ensure their values and treatment preferences are upheld. This case highlights a geriatric patient with a complex medical history who outlined his wishes before his condition was compromised. By initiating these conversations prior to medical decline, healthcare workers can respect the patient’s wishes and reduce the emotional burden on family members. They were able to appoint a medical POA and develop a living will. Effective ACP not only reduces unnecessary interventions and hospitalizations but also improves the patient’s psychological well-being and quality of life moving forward. The context of this patient brings an interesting perspective on ACP, especially since the patient was admitted to the hospital for his hallucinations but ended up deceasing during that same hospital stay from respiratory failure. Without proper education about ACP, this patient could have received undesired interventional treatment during his stay. This case reinforces the value of individualized end-of-life care that incorporates ACP as a preventative and forward-thinking strategy as opposed to last-minute responses in geriatric management.

## Introduction

In terms of end-of-life planning and medical decisions, approximately 69% of geriatric patients (> 65 years old) establish advance directives (ADs) and about 72% are likely to delegate a power of attorney (POA) in their lifetime [1]. This leaves around 30% of patients to pass away with unknown desires of care [2]. Advance care planning (ACP) allows patients to articulate (verbally and physically on paper) their healthcare values and desires before they are incapable of making those decisions themselves. ACP can be seen through verbal discussions with physicians, lawyers, or family members as well as written documents to express desires on paper [3]. Despite the many benefits these documents provide, only 37% of adults in the United States complete an AD prior to mortality whereas older patients above the age of 65 show slightly higher participation around 45% [4]. Specific end-of-life documents include, but are not limited to, living wills, POA for healthcare, and POLST which guide care teams when faced with end-of-life decisions and treatment [5]. Additional advance planning forms available for consideration are do not resuscitate (DNR), do not intubate (DNI), do not hospitalize (DNH), and other lesser-known life-sustaining treatment forms such as the medical order for life-sustaining treatment (MOLST) and an out-of-hospital DNR order [5].

In older patients, especially those with dementia or delirium, these documents are crucial due to compromised decision-making skills and mental function [6]. Addressing healthcare needs as soon as possible can remove the stress placed on family members as they are forced to make medical decisions on behalf of the patient [7]. In the long run, family members have less concern regarding the patients’ desires with a detailed outline of the actual wishes [8]. A study confirms the benefits of ACP for the well-being of family members, stating that they had significantly less stress, anxiety, and depression when the patient’s wishes were outlined before death [7].

Common reasons for not creating ADs are lack of trust between the patient and relatives/physicians, lack of interest, and lack of guidance [1]. Despite the challenges, creating these legal documents is vital for patients, especially older ones, to outline their preferences for the future of their health, assets, and finances as well. Without these outlined wishes, individual state laws delineate decision-making power to a default individual; usually the patient’s partner, but oftentimes adult children or friends are appointed [5]. ACP, although it is most commonly utilized in older and terminally ill patients, can prevent unwanted treatment of the patient if the desires and decision makers are priorly assigned at an early age [9]. A study done by Rao et al reflects that younger patient populations have a severely low rate of AD completion: 11.8% of ages 18–34, 19.2% of ages 35–54, and 29.3% of ages 55–65 [10]. This is likely due to younger populations not understanding the value or relevance of ACP.

In patients who present with multiple comorbidities, a goals of care (GOC) conversation is recommended to help decide what matters most to the patient themselves. These conversations can look different depending on what stage of life the patient is in; early conversations can consist of future treatment options, and later conversations can discuss interventional techniques that are necessary to keep the patient alive, such as DNR/DNI orders as well as cardiopulmonary resuscitation (CPR) [11]. This case report involves a patient with multiple comorbidities: Charles Bonnet syndrome (CBS), chronic obstructive pulmonary disease (COPD-stage unknown), diabetes mellitus, hypertension, chronic kidney disease (CKD), etc. and highlights the importance of ACP strategies which were planned before future complications. Planning is vital for comorbid patients due to the unexpected nature of treating some conditions without worsening others.

## Case Report

After reaching out to the family, consent was received to present this patient’s case. A 92-year-old male patient with a history of diabetes mellitus, with HbA1c value of 8.9, stage 3 CKD, hypertension, COPD, chronic hypoxemic respiratory failure, multiple wounds to bilateral lower extremities, and morbid obesity presented to the emergency department for evaluation of hallucinations presented as flowers, bees, and bugs, along with worsening conditions following a fall on a week prior. The patient reported left knee pain following knee replacement surgery alongside a ground-level fall, shortness of breath, and a cough. The patient is a former cigarette user with 53 pack-years. He requires 4 L of oxygen, due to hypoxemia from COPD, at home where he lives with his son and daughter-in-law. He has no history of past mental illness or cognitive dysfunction. Current medications at time of admission include over-the-counter cetirizine, melatonin, vitamin D3 supplement, and prescription medications including an albuterol inhaler and furosemide.

Following admission to the inpatient hospital medicine service, psychiatry was consulted to do a workup on the visual and auditory hallucinations present for the past 3 days. During the first consultation, the patient reported hallucinating and seeing flowers and birds, along with hearing bees buzzing. However, the patient remained alert and oriented to his location, person, and time. At the second consultation, he still reported seeing bugs, rats, and spiders. He denied any past psychiatric history and medications. After verbal questioning, psychiatry eliminated primary psychiatric disorders from lack of mental decline indicators such as change in mood, anxiety, depression, etc. To establish potential cognitive causes of hallucinations, a computed tomography (CT) of brain was ordered and reflected mild white matter disease and asymmetric density near the left choroid. Following the CT scan findings, at this point, possible diagnoses considered were delirium, cerebrovascular accident (CVA), migraine, infection, and CBS. As part of the differential diagnoses, a chest X-ray was performed, which revealed an elevation of the right hemidiaphragm, hence, a CT pulmonary scan was ordered, revealing mild interstitial pulmonary edema and patchy bibasilar opacities suggestive of atelectasis or early onset of pneumonia.

On day 4 following admission, rapid response was called as the patient was experiencing foaming at the mouth and had a grey appearance, likely from hypoxemia. He received oxygen support and transitioned to a Venturi mask. A pulmonary embolism was suspected as the cause, but further testing and imaging was declined by his POA—his family. In compliance with the patient’s POA and POLST, they opted for comfort measures and DNR/DNI orders. He directed his POA to his son, along with two additional familial signees who made the ultimate decision to reject any invasive treatment and place the patient on comfort care precautions. Hospice services had been arranged previously, and the patient was awaiting long-term care placement when he experienced this terminal respiratory decline. The patient passed with presumptive cause of death being respiratory failure, secondary to aspiration pneumonia.

## Discussion

This case outlines the importance of ACP leading up to mortality, specifically in geriatric patients with complex comorbidities. This patient presented with complex respiratory, neurological, and internal illnesses. This patient’s conditions were complex, but not rare. Despite the patient’s conditions being fairly common, it is still vital to consider the steps he took in his health journey to inform others who might go through a similar scenario. To prepare, he compiled a POA and POLST, delegating his son with medical decision-making power and outlining his wishes for DNR/DNI and an overall comfort care plan. With this patient’s complex medical history, it was inevitable that the family would be left with tough decisions regarding end-of-life care. Therefore, the patient outlining these wishes prior to his complications removed a burden from the family members who would have had to make an ultimate decision when he was sustaining end-stage respiratory failure. A comparison can be made with the 2001 California Supreme Court case Conservatorship of Wendland, when patient Robert Wendland became incapacitated following complications from brain surgery [12]. Due to his state of health, his wife sought to remove his feeding tube; however, in the absence of explicit documentation, his care preferences remained unclear [12]. This not only led to the wife being forced to argue with medical staff but also led to profound distress, uncertainty, and emotional burden on the spouse/family members [12]. This problem could have been simplified with proper ACP discussions and documentation, allowing both parties to focus on the facts instead of jumping to assumptions. A linkage between geriatric patients with multiple comorbidities, including CBS, and ACP is limited; however, this case presents a patient with proper preparation techniques.

ADs have been shown to improve patient satisfaction (17% increase in quality of visit from patients without advanced directive experience) [13]; however, there is an ongoing debate on the moral justifications of life-prolonging treatments to terminally ill patients. Artificial prolonging of life was debated between Crippen and Hawryluck which describes the benefits and drawbacks of life support for a particular respiratory failure patient. Medical professionals believed this patient was not well suited to survive in an intensive care unit (ICU) [14]. Without ADs from the patient, there is not a clear answer as to if medical ventilation was within the boundaries of the patient. The debate was between the premise of the law and its implications, whereas the other side is that ventilation is not an ultimate solution or cure, deeming it pointless [14]. In terms of CPR, there is an immediate survival rate of 37-61% in adult patients; however, only 25% of them end up getting discharged from the hospital before mortality [15]. This proves a relatively low long-term benefit but can still aid depending on the quality of the patient’s outcome. Life support not only takes emotional tolls on families, but financial burdens as well. On average, a family can spend around $100,000/year to maintain life support functions when the yearly mortality rate is 50% in ill patients over the age of 65 [16].

The uplifting aspects of end-of-life planning are vast, with a few including: boosting confidence in honoring wishes, prior discussion which is helpful for real-time decisions, greater sense of preparedness, perceived lack of alternatives when medical outcomes seem unavoidable, and lingering emotional struggles when experiencing doubt/remorse towards the situation [17]. Not only are there benefits on the family side, but also on the outcome of the patient themselves, with a higher survival rate in many circumstances. Furthermore, patients with a living will have 38.8% likelihood of deceasing during hospitalization, whereas that risk increases to 50.4% for those without documentation—an overall 11.6% increase [18]. These findings highlight the importance of ACP, as clearly stated wishes can reduce uncertainty and prevent unnecessary/undesired treatment, ultimately resulting in better outcomes for both patients and their families.

Given this patient’s extensive medical history of chronic conditions such as COPD, diabetes, and obesity, mechanical ventilation may have temporarily resolved his respiratory function, but it would not have addressed the broader decline in his health. His multiple comorbidities contributed to a complex dynamic of intervention treatment, including intubation, which could have prolonged his life briefly without providing progress in his overall prognosis. Evidence suggests that hospitalized patients with three or more comorbidities face higher mortality rates (56.5%) compared to the mortality rate in those with fewer than three comorbidities (33.5%) [19]. This is important to consider since this patient had greater than three comorbidities, therefore putting him more at risk. In this scenario, life support treatments risk becoming more harmful than beneficial, underscoring the importance of aligning patient care with overall goals.

The research merging the topics of ACP with multimorbid geriatric patients is limited. Despite this gap in literature, many studies have focused on multimorbid effects on COVID-19 patients specifically, and the most commonly seen: diabetes, hypertension, cardiovascular diseases, obesity, and cancer [20]. These data add to the severity of complex comorbidities seen in this case study of this patient from the overlap of diabetes, hypertension, and obesity. Considering the risks of the pandemic alongside preexisting conditions, literature has established the need for ACP in multimorbid patients. These studies compile the relationship between comorbidities and complications; however, a commonly overlooked aspect is mental decline with aging.

Although there is limited evidence between ACP and multimorbid geriatric patients, this case report compiles evidence from various realms to prove the importance of end-of-life planning. Without the consideration of this case report, there is a lack of emphasis on ACP. This case is important to consider on a worldwide standpoint as a standard for individuals to follow. No matter their background or location, ACP can be useful for individuals beyond the United States.

### Conclusion

Designating a medical POA provided the patient with a trusted advocate during his end-of-life treatment. Without proper guidelines laid out prior to his death, his wishes may not have been upheld to his liking. This case emphasizes how a proactive approach to ACP can advance decision-making preparation, especially in complex geriatric presentations where nonfatal diagnoses, like CBS, may mask deeper clinical needs. Initiating ACP discussions early on can enable patients and families to be on the same page regarding goals and desires of medical treatment. This case is additionally relevant to healthcare professionals in order for them to provide the best individual care to their patients.

## Data Availability

The authors declare that data supporting the findings of this study are available within the article.

## Abbreviations

ACP: advance care planning
AD: advance directive
CBS: Charles Bonnet syndrome
CKD: chronic kidney disease
COPD: chronic obstructive pulmonary disease
CPR: cardiopulmonary resuscitation
CVA: cerebrovascular accident
DNH: do not hospitalize
DNI: do not intubate
DNR: do not resuscitate
GOC: goals of care
MOLST: Medical Order for Life-Sustaining Treatment
POA: power of attorney
POLST: Physician Orders for Life-Sustaining Treatment
